# Multidomain interventions for sarcopenia and cognitive flexibility in older adults for promoting healthy aging: a systematic review and meta-analysis of randomized controlled trials

**DOI:** 10.1007/s40520-024-02700-2

**Published:** 2024-02-22

**Authors:** A. M. García-Llorente, A. J. Casimiro-Andújar, D. G. Linhares, R. G. De Souza Vale, P. J. Marcos-Pardo

**Affiliations:** 1 Present Address: SPORT Research Group (CTS-1024), Department of Education, CIBIS (Centro de Investigación Para el Bienestar y la Inclusión Social) Research Center, Area of Physical Education and Sport, University of Almería, Office 0.22, Building CITE III, Almería, Spain; 2 Department of Education, Faculty of Education Sciences, University of Almeria, 04120 Almeria, Spain; 3 Active Aging, Exercise and Health/HEALTHY-AGE Network, Consejo Superior de Deportes (CSD), Ministry of Culture and Sport of Spain, 28040 Madrid, Spain; 4 Universidade do Estado de Rio de Janeiro, Rio de Janeiro, BR Brazil; 5 Laboratorio de Ejercicio y Deporte, Instituto de Educación Física y Deportes, Universidad del Estado de Rio de Janeiro, Rio de Janeiro, 20550-900 Brazil

**Keywords:** Aging, Multidomain, Sarcopenia, Cognitive flexibility, Meta-analysis

## Abstract

The global population is undergoing rapid aging, making physical activity a crucial element in preventing mortality and lowering the risk of Alzheimer's disease. Age-related declines in muscle mass and cognitive abilities significantly contribute to frailty and dependency. Thus, this study focuses on a meticulous analysis of the protective effects of multidomain interventions, an emerging resource combating age-related declines. It seeks to underscore their profound impact on cognitive flexibility and sarcopenia, highlighting their pivotal role in mitigating the adverse effects of aging. To identify relevant randomized controlled trials up to November 2023, we reviewed eight online academic databases, following PERSiST guidelines, PRISMA reporting system, and PICOs criteria. Meta-analyses on selected functional outcomes utilized a random-effects model, including the Timed Up and Go Test, Sit to Stand Test, Victoria Stroop Test, and Trail Making Test. Out of 2082 scrutinized articles, 17 were included in the systematic review, and 8 in the meta-analysis. Positive effects (*p* = 0.05, *I*^2^ = 57%; 95% CI – 0.63 to – 0.05) were observed in cognitive flexibility for certain interventions. Similarly, interventions addressing muscle strength demonstrated improvements in the Sit to Stand Test for the exercise group compared to the control group (*p* = 0.02, *I*^2^ = 0%; 95% CI – 0.63 to – 0.05). These findings underscore the importance of incorporating physical activity as a primary component of public health interventions for promoting healthy aging and reducing the burden of age-related diseases. Future interventions may explore more homogeneous approaches and evaluate the impact of thrice multidomain weekly sessions.

**Supplementary Information:**

The online version contains supplementary material available at 10.1007/s40520-024-02700-2.

## Introduction

### Rationale

The global aging population poses both challenges and opportunities for societies worldwide. With increasing life expectancies and declining birth rates, the proportion of older adults in the global population is growing at an unprecedented rate. According to the World Health Organization (WHO), it is projected that by 2050, the number of people aged 60 years and above will reach 2 billion, constituting approximately 22% of the global population [1]. Additionally, the burden of physical inactivity exacerbates this situation [2]. This demographic shift and the attitude toward physical activity necessitate a comprehensive understanding of the factors influencing healthy aging to effectively address the associated challenges.

A critical aspect of healthy aging is frailty, representing a complex state of vulnerability that increases the risk of adverse health outcomes in older adults [3, 4]. Frailty encompasses various dimensions, including physical, psychological, and social aspects. Determinants influencing frailty, notably sarcopenia and cognitive decline, are pivotal elements in the distressing trajectory leading to dependency. Sarcopenia, the age-related loss of muscle mass and strength, significantly contributes to the development toward dependency. Emerging evidence suggests the importance of regular physical activity, resistance training, adequate diet, and sleep in preventing and managing sarcopenia [5, 6]. Strategies aimed at maintaining muscle health, particularly resistance training, should be incorporated into interventions targeting healthy aging. Furthermore, sarcopenia is strongly associated with cognitive impairment in older adults [7].

Cognitive decline, which includes impairments in memory, attention, and executive functions, is another critical aspect affecting healthy aging. Cognitive flexibility, the ability to adapt cognitive processes in response to changing circumstances, plays a vital role in maintaining cognitive function and overall well-being in older adults [8]. Enhancing cognitive flexibility through cognitive training and engaging in intellectually stimulating physical activities, such as dual-task physical–cognitive training, may mitigate cognitive decline. Physical activity interventions have also been shown to be effective in preventing one-third of Alzheimer's and dementia clinical cases [9]. Additionally, cognitive flexibility is strongly associated with mild cognitive diseases (MCD) and the cognitive decline of the population [10].

Promoting healthy aging requires a multidimensional approach that addresses frailty, sarcopenia prevention, cognitive decline, and more specifically cognitive flexibility. The global situation of an aging population necessitates comprehensive strategies to effectively enhance the well-being and quality of life of older adults. Over the last decade, there has been increased interest in the influence of exercise on broad executive functions [11]. However, there is limited information about how multidomain interventions may influence both cognitive flexibility and sarcopenia. While Xiong and colleagues recently studied the influence of different types of exercise on executive functions [11], they did not reflect on sarcopenia, which we consider essential in the aging process. The primary objective of this meta-analysis is to scrutinize the existing evidence available for interventions addressing sarcopenia and cognitive flexibility. This novel approach endeavors to formulate an effective strategy aimed at enhancing the quality of life for the elderly population, with a particular emphasis on mitigating factors that may lead to dependency.

Given the multifaceted dynamics inherent in healthy aging, interventions concurrently addressing multiple domains have garnered significant attention. Multidomain interventions integrate strategies targeting physical activity, cognitive stimulation, social participation, etc. [12, 13]. Notably, multidomain interventions incorporating dual tasks for both physical and cognitive training, show promise in fostering healthy aging and averting frailty-related adverse outcomes [14, 15]. In the context of this study, a dual task will be considered as the simultaneous training of physical and cognitive aspects, with a particular exercise, involving one or more tasks within the multidomain intervention. Consequently, multidomain interventions including strength training, cardiorespiratory training, cognitive stimulation, and social engagement may enable stakeholders to address the various factors influencing healthy aging, thereby improving the overall well-being for older adults.

In relation to this meta-analysis, the duration cut-off was 8 weeks. According to the position statement from the national strength and conditioning association (NSCA) for older adults, effective strengthening sessions should be of a duration no less than 6 weeks [16]. However, they have been deemed insufficient to produce a reduction in inflammation measured with C‐reactive protein and TNF-α.

## Methods

This study was conducted in line with the PERSiST guidelines for systematic reviews [17], a sports and exercise medicine alignment of the 27 PRISMA statement [18]. It was also prospectively registered in the international database for reviews from the National Institute for Health Research (NIHR), Prospero, under the reference number (*CRD42023400224*).

### Eligibility criteria

*Inclusion criteria* 1.Complete original study; 2.Clear intervention; 3.Randomized Controlled Trials (RCT); 4.≥ 65 years old physically independent; 5.The intervention addressed both sarcopenic/strength training and cognitive flexibility outcome measures. Any studies that examined other chronic effects, alternative training methods, or disease specific studies were not included.

*Exclusion criteria* 1.Insufficient length of the intervention (< 8 weeks); 2.Not written in English, Spanish, Portuguese or French; 3.Absence of a passive control group; 4.Institutionalized patients (care homes, hospitals, etc.); 5.Studies including nutritional supplementation.

### Information sources

A systematic literature search was conducted by two independent investigators between April and November 2023. The search was carried out using the following online databases to identify relevant articles: PubMed, Scopus, Cochrane, Science Direct, PEDro, Web of Science, EBSCO, and Nature. Furthermore, the reference lists of all included studies were scrutinized to find other eligible papers, which resulted in the inclusion of gray literature.

### Search strategy

We conducted a search using specific terms related to the intervention. These terms were combined with Mesh terms to enhance the accuracy of our results. Our search criteria were as follows: we included terms such as ("Executive Function" OR "Cognitive Flexibility") AND (Multidomain OR Multi-domain OR Multicomponent OR "Dual task") AND (Sarcopenia OR Strength* OR "Resistance training"). To ensure comprehensive coverage, we adapted these search terms for other bibliographic databases, using database-specific filters for controlled trials when available (*Annex A*). In addition to the database searches, we reviewed the authors' files and examined the reference lists of each included article. Prior to conducting the final analysis, we repeated the searches and identified additional studies for potential inclusion. There was a time criterion for the last 10 years set from December 2012 until the end of the screening period, November 2023. Titles and abstracts that did not meet the predetermined inclusion criteria were subsequently excluded from the list.

### Selection process

The literature search strategy for this study followed the PRISMA guidelines, which provide a standardized approach for reporting systematic reviews and meta-analyses. These guidelines ensure that all essential elements are included in the report. Additionally, the search strategy incorporated the Problem/Population, Intervention, Comparison, Outcome, and Study (PICOs) framework. The PICOs framework helps formulate key questions that guide the search for high-quality evidence effectively.

*Population:* Older adults aged > 65 living independently; *Intervention*: Multidomain, multicomponent or dual-task training of at least 8 weeks; *Comparator*: Passive control group (absence of cognitive or physical training); *Outcome measures*: Sarcopenia and cognitive flexibility. Including Timed Up and Go (TUG), Sit to Stand Test (STS), Trail Making Test (TMT), and Victoria Stroop Test (VST); *Study*: Randomized control trials.

Considering the high degree of heterogeneity in reporting the results from the study selection, we decided to exclude some articles from further analysis and the reasons can be found below. However, they were still included in *Tables 1, 2, and 3* (*Annex B, C, and D).*

### Data collection

The search for relevant studies on a specific topic was conducted by two independent authors. The screening process consisted of four stages: First, the reviewers evaluated the titles of the studies to determine their suitability for inclusion in our meta-analysis. Second, the abstracts of the selected titles were assessed to ensure that the study topics met the predetermined inclusion and exclusion criteria. Third, the full-text articles were thoroughly examined using relevant keywords, and the articles deemed relevant were then uploaded to Mendeley. Lastly, the references of the included studies were carefully reviewed, and studies that did not provide the necessary information, such as outcome measures, were excluded. In cases where there were disagreements regarding the inclusion or exclusion of certain RCTs, resolution was achieved through discussion or involvement of a third party when consensus could not be reached.

### Data items

The study items were divided in three sections: 1.Study characteristics: including the author's name, country where the intervention took place, sample size (expressed in mean and standard deviation), and the percentage of female participants in both the experimental group and control group. In instances where studies featured multiple experimental groups, the data were documented when the relevant outcome measures were observed in the post-evaluation. 2.Additionally, study intervention encompassed details such as the type of intervention, total duration of the session, training volume (comprising session duration, session frequency, and total number of sessions during the intervention), dose/intensity (encompassing intensity level and measurement methodology for each training type), and supplementary information that could have potentially impacted the study results. 3.Finally, the relevant outcome measures were identified, and the post-evaluation results were reported using either mean and standard deviation values or confidence intervals (CI), along with their corresponding levels of significance. Only the outcomes related to the study were included in the Table of results.

### Risk of bias assessment

To assess the risk of bias (ROB), the researchers used the RoB 2 [19], a framework that serves as a guide for evaluating the potential for bias in the results of randomized trials. It focuses on assessing the relative effect of two interventions or intervention strategies on a specific outcome in a single trial. These interventions are referred to as the experimental intervention and comparator intervention, though the comparison may sometimes involve two active interventions. The framework consists of five domains, which were determined based on both empirical evidence and theoretical considerations, that identify how bias may be introduced into the result. The five domains are: 1.bias arising from the randomization process; 2.bias due to deviations from intended interventions; 3.bias due to missing outcome data; 4.bias in measurement of the outcome; and 5. bias in selection of the reported result.

### Assessment of methodological quality

To assess the methodological quality of the study, the researchers used the Tool for the Evaluation of the Quality of Study and Report in Exercise (TESTEX) [20], which is specifically designed for physical exercise studies. TESTEX consists of a scale that includes criteria for evaluating internal validity and the statistical analysis presented in experimental studies. Each indicator defined in the scale is assigned one point, and zero points are given in the absence of these indicators. The criteria in the scale include: 1.specification of inclusion criteria; 2.random allocation; 3.allocation secrecy; 4.similarity of groups in the initial or baseline phase; 5.blinding (for at least one key outcome assessed); 6.measure of at least one primary completion in 85% of the allocated subjects (up to three points); 7.intention-to-treat analysis; 8.comparison between groups of at least one primary dropout (up to two points); 9.report measures of variability for all reported outcome measures; 10.monitoring of activities in control groups; 11.the relative intensity of constant physical exercise; and 12.characteristics of exercise volume and energy expenditure.

### Effect measures

According to the European consensus on the definition and diagnosis of Sarcopenia [6], the parameters for defining sarcopenia are best characterized by muscle strength assessments, such as the STS test, muscle quality evaluations, and physical performance measures like the TUG. Furthermore, recent research indicates that muscle strength serves as a more reliable indicator of poor cognitive function compared to other markers such as lean mass [21].

In the context of measuring cognitive flexibility, a commonly used tests in the older population include TMT. However, it is worth noting that the level of education among participants may have an influence on the test results [22], thus warranting further investigations to account for this potential confounding factor.

### Reporting bias assessment

In the assessment of reporting bias, three articles were excluded due to bias risk or methodological reasons: The articles [23–25], were excluded from the selection process due to a substantial risk of bias. This decision was made to ensure the reliability and validity of the study's findings. Similarly, an additional manuscript [26] was ruled out due to methodological reasons, specifically the high volume of dropouts observed in the control group, based on the TESTEX methodological risk (*Annex E*). By excluding this study, the researchers aimed to maintain the integrity of the control group and minimize potential confounding factors, resulting the selection in 17 articles for the systematic review [12, 27–42].

### Meta-analysis

The Review Manager 5.4.1 program (RevMan version 5.4.1; The Cochrane Collaboration, Oxford, UK, available at (http://tech.cochrane.org/revman) was used to analyze muscle power (STS), functional strength (TUG), cognitive function (VST), and cognitive flexibility (TMT). Meta-analyses were performed when two or more studies could be pooled based on similar interventions and the same outcome variables assessed with the same tests. Each standardized mean difference (SMD) was weighted according to the inverse variance method. The SMD values in each study were pooled with a random, if heterogeneity was significant model, or fixed effect if heterogeneity was by chance. Such model is used when heterogeneity is low (*I*^2^ < 0.50). However, when there is a moderate or high degree of heterogeneity (*I*^2^ ≥ 0.50) between studies, the random effect model should be considered. SMD values were interpreted as: < 0.2: weak; 0.2–0.79: moderate; ≥ 0.8: strong [43]. A statistically significant effect was indicated by *p* < 0.05.

### Evidence-level assessment

Two authors independently assessed the certainty of evidence using the Grading of Recommendations Assessment, Development and Evaluation (GRADE) approach with the GRADE PRO website, available at https://gradepro.org. GRADE specifies four categories: “high,” “moderate,” “low,” and “very low,” applied to a body of evidence. RCTs begin with high-quality evidence. Five aspects can decrease the quality of evidence: methodological limitations, inconsistency, indirect evidence, inaccuracy, and publication bias. On the other hand, three aspects can increase the quality of the evidence: effect size, dose–response gradient, and confounding factor [44]. Heterogeneity between studies was analyzed using *I*^2^ statistics. *I*^2^ values are interpreted as low heterogeneity (0–50%), moderate heterogeneity (50–74%), and high heterogeneity (≥ 75%) [45, 46] (*Annex F*).

## Results

### Search results

**Fig. 1:**
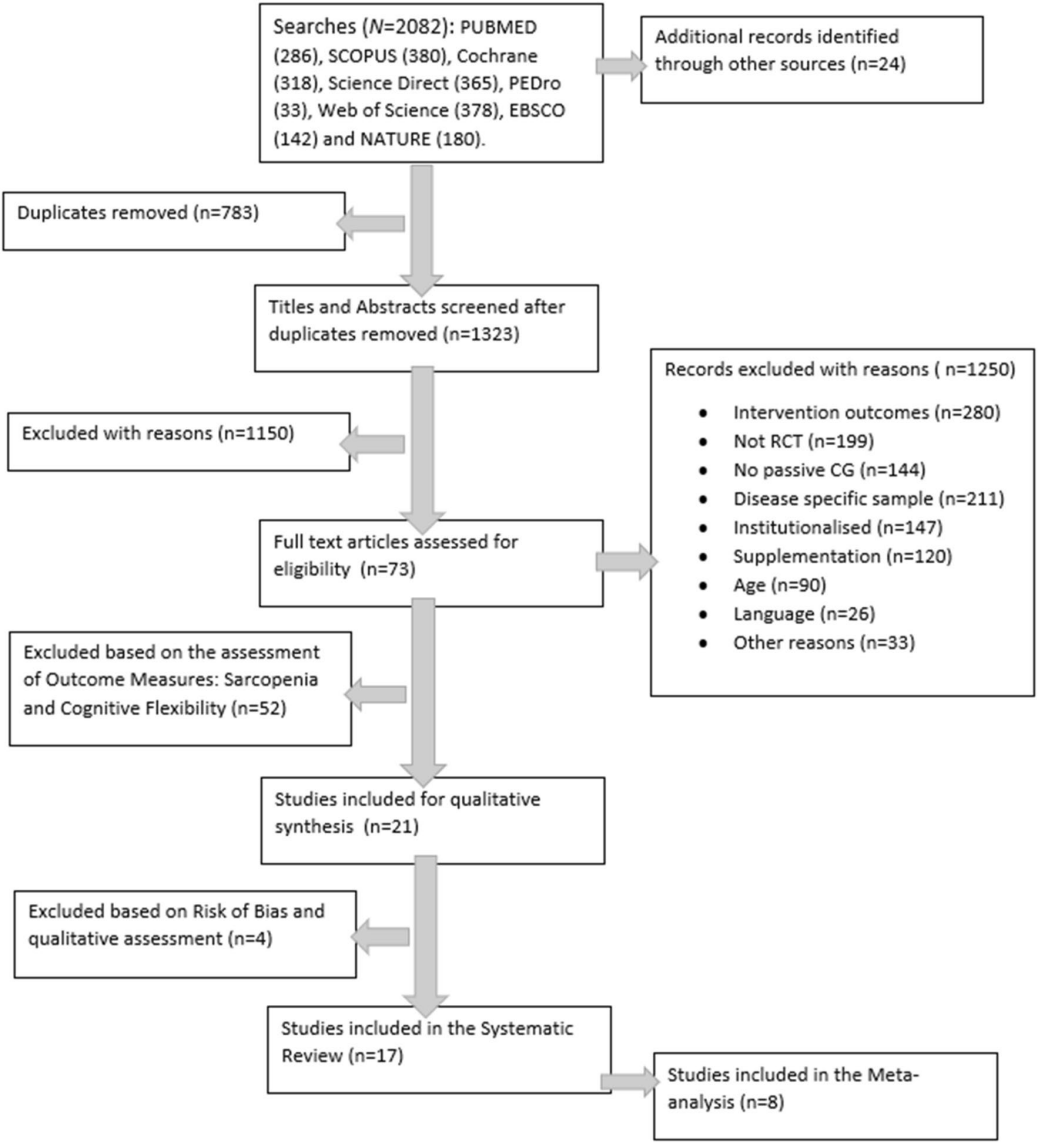
Flow diagram of the studies´ selection process

The initial search yielded a total of 1982 studies, of which 783 duplicates were eliminated. Moreover, 24 studies from alternative sources were added. After careful consideration, 1151 records were excluded for distinct reasons. Subsequently, 71 studies underwent thorough full-text review, and 21 RCTs proceeded to the assessment stage for bias evaluation. Ultimately, a total of 17 articles were included in the systematic review, and among them, 8 were used for the meta-analysis [12, 27–30, 32, 37, 39] (Fig. 1).

### Study selection

Once the duplicates were removed, two researchers individually reviewed the selected titles. In the event of a disagreement, a third researcher would help to reach consensus. Additionally, to take into consideration gray literature, the reference lists of primary selected studies and related systematic reviews and meta-analysis were reviewed.

The following studies were included in the systematic review but not in the Meta-analysis for the reasons below: (A) The studies contained mean differences between pre-tests and post-tests but no information about post-test results [35, 39]; (B) The relevant outcome measures for sarcopenia and/or cognitive flexibility were not included in other studies, so a meta-analysis could not be performed.

### Study characteristics

*Table 1* displays the characteristics of the studies included in this review. The selection process encompassed a global representation, resulting in 17 studies (10 from Asia, 4 from Europe, 2 from Australia, and 1 from South America. All studies had female participants, with the experimental groups comprising at least 41.3% females, except for the study conducted by Wang and collaborators [41]. The sample sizes of the studies varied, ranging from 19 participants [33] to 309 participants [35], resulting in a total sample size of 1036 participants across all studies. The studies selected focused on older adults with average ages surpassing 70 years old, with the exceptions being the studies by Coelho and collaborators [32], Iuliano and colleagues [36], and Kim and colleagues [38]. Notably, none of the included studies reported any adverse events associated with the interventions or interventions' outcomes.

### Meta-analysis results

**Fig. 2:**
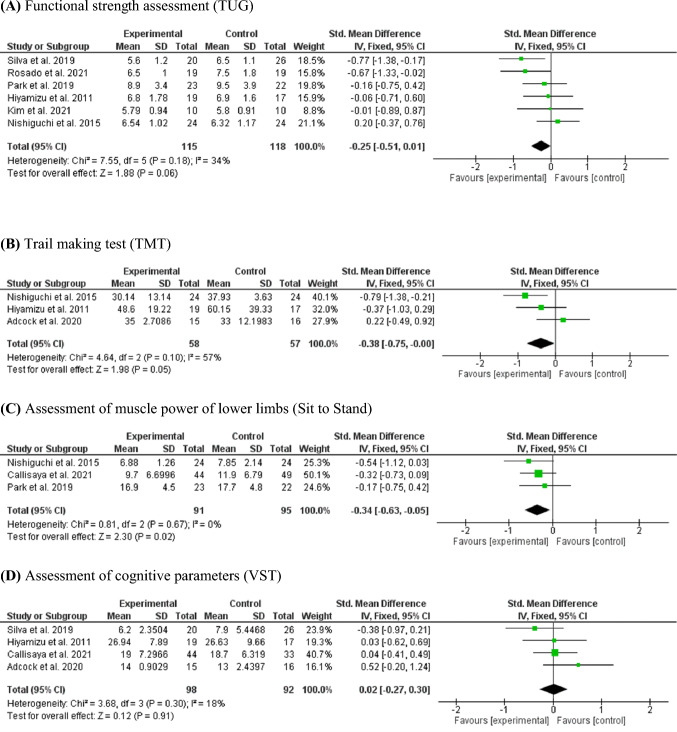
Forest plot TUG (**A**), TMT (**B**), Sit to Stand (**C**), and VST (**D**). **A** Functional strength assessment (TUG). **B** Trail making test (TMT). **C** Assessment of muscle power of lower limbs (Sit to Stand). **D** Assessment of cognitive parameters (VST)

Figure 2 presents the results of the meta-analysis of the studies that investigated the variables of muscle power, functional strength, cognitive function, and cognitive flexibility. The effect size was calculated by SMD with a CI of 95%. When calculating the effect size, the negative sign means greater effects for the exercise group (EG) when compared to the control group (CG). The diamond represents the average effect size of the included studies and should be interpreted equally.

Figure 2A presents the results of the meta-analysis of studies that used the TUG in the assessment of functional strength. There was no significant improvement; however, the presented p-value is close to results that indicate a trend toward improvement in this variable (functional strength) (95% CI – 0.51 to 0.01) in favor of EG participants with inconsistency *I*^2^ = 34% and *p* = 0.06.

Figure 2B shows the results of the meta-analysis of studies that used the TMT to assess cognitive flexibility. There was no significant difference in TMT (95% CI – 0.75 to 0.00) in between participants in the EG and CG groups with *I*^2^ inconsistency = 57% and *p* = 0.05. The results indicate a trend toward improvement in this variable in favor of EG.

Figure 2C shows the results of the meta-analysis of studies that used the STS to assess lower limb power. There was a significant difference in Sit to Stand (95% CI – 0.63 to – 0.05) in favor of EG participants with inconsistency *I*^2^ = 0% and *p* = 0.02.

Figure 2D shows the results of the meta-analysis of studies that used the VST to assess cognitive parameters. There was no significant difference (95% CI – 0.27 to 0.30) between participants in the EG and CG groups with *I*^2^ inconsistency = 18% and *p* = 0.91.

Eight of the 17 studies were meta-analyzed, with Hiyamizu, Nishiguchi, Adocock, and collaborators [12, 27, 28] showing a strong tendency for improvement in the TMT variable (*p* = 0.05). The VST variable did not exhibit differences (*p* = 0.92). The STS variable indicated increased muscle strength in the exercise group compared to the control group (*p* = 0.02) [28–30]. There was a potential improvement in functional strength (TUG test) [27–29, 32, 33, 38], with values approaching statistical significance (*p* = 0.06). Caution is warranted in interpreting these results due to the limited number of meta-analyzed studies per variable and the low heterogeneity.

## Discussion

The primary aim of this meta-analysis was to assess the impact of multidomain exercises on sarcopenia and cognitive flexibility in older individuals. The VST test was utilized for cognitive analysis, with interventions ranging from resistance training to multidomain exercises, exergame, and aerobics. Notably, some studies demonstrated a significant improvement in cognition (*p* < 0.05) [12, 36, 37, 42]. These findings align with Chaparro et al.'s cross-sectional study [47], which indicated improved cognition with specific interventions. These results are consistent with those of the Framingham study [48], emphasizing the cognitive benefits of exercise, particularly in Alzheimer's patients [49].

However, when examining cognitive flexibility through the TMT test [12, 27–29, 36], no significant improvements were observed despite interventions involving exergame and multidomain exercises. Imaoka et al.'s study [50] also found no differences in cognitive flexibility (*p* > 0.05) in older individuals undergoing multidomain exercise. The insufficient duration and intensity of the interventions might explain these outcomes, underscoring the importance of adhering to WHO recommendations for physical activity [1].

Muscle strength, evaluated through the Sit to Stand Test (STS) [27–30, 32] and HGS-R test [29, 50], showed varied results. While some studies reported increased muscle strength (*p* < 0.05), others, such as those by Calisaya et al. and Hiyamizu et al., did not observe significant changes (*p* > 0.05). Divergent outcomes may be attributed to differences in training volume, intensity, frequency, and load control.

Functional strength, assessed by the TUG test [27–29, 31, 32, 36, 37], exhibited improvements in some studies (p < 0.05), suggesting that multidomain exercise prescriptions can enhance the functional strength of older individuals, contributing to fall prevention and increased independence. Corroborating these findings, Sadjapong and collaborators [51] used multidomain exercises in 2 groups (multidomain x usual activities), with a frequency of 2 × week for a total period of 24 weeks in 64 older people with a mean age of 78 years. In this sense, Sanders, and colleagues [52] analyzed 69 older people comparing 2 groups of exercises at different intensities with a frequency of 3 × week for 24 weeks (low intensity x high intensity exercises). The authors did not find improvements (*p* > 0.05) in muscle strength. The divergent results reported may be due to the intensity of training, weekly frequency, and load control.

Cardiorespiratory fitness, measured through the walking test (WT) [28, 30, 31], demonstrated increased capacity in most studies (*p* < 0.05), with variations attributed to differences in intervention types. The SPPB battery revealed mixed results [36, 37], emphasizing the potential influence of exercise intensity on functional fitness.

Body composition assessments (% Fat and MM) [37, 38] showed varied outcomes. Kim et al.'s study reported an increase in MM, while Park et al.'s study [53] demonstrated improvements in body composition (% fat and BMI). The impact of exercise intensity on physiological adaptations was evident.

Noteworthy is the reduced number of weekly training sessions in some studies [28, 29], potentially influencing results in functional strength assessment (TUG test). Increased weekly sessions may generate positive effects on physical fitness due to chronic physiological adaptations [16]. It is recommended to consider variables such as weekly frequency, session duration, intensity, volume, exercise type, and total intervention period for effective exercise program prescriptions for older individuals.

### Limitations

The limitations of the present study were as follows: First, the small number of studies included in the meta-analysis per variable. Second, it may be advisable for future studies to explore the retention of cognitive and physical improvements over time. It is unknown whether the positive effects multidomain interventions have on older individuals are retained over time. Finally, future interventions may benefit from using the Latin American Group for Maturity battery (GDLAM) [54] which offers improved property in determining the risk of sarcopenia as opposed to SPPB based on the European Working Group on Sarcopenia in Older People guidelines (EWGSOP19) [6].

## Conclusions

Multidomain interventions that encompass both cognitive and physical training for at least 8 weeks within a training plan have been shown to be effective in improving both muscle strength and cognitive flexibility among older adults. In addition, multi-dominance training also has the potential to improve muscle function and balance. Consequently, multidomain training may contribute to the prevention and treatment of age-related diseases, including cognitive diseases such as Alzheimer's disease. Based on this novel information, policy makers, health professionals, and researchers can develop and implement effective non-pharmacological intervention strategies with multidomain interventions to promote healthy aging and ensure societies successfully adapt to the challenges and opportunities presented by a rapidly aging world.

### Supplementary Information

Below is the link to the electronic supplementary material.

Supplementary file1 (DOCX 201 KB)

## Data Availability

Annex A in appendices shows where the information can be found. Pre-register of the study: the study was uploaded to **Prospero** with the ID number (*CRD42023400224*).
