# Supplementary material for: Multidomain interventions for sarcopenia and cognitive flexibility in older adults for promoting healthy aging: a systematic review and meta-analysis of randomized controlled trials
Source: Aging Clin Exp Res. 2024 Feb 22;36(1):47. doi: 10.1007/s40520-024-02700-2 (PMC10884056; doi:10.1007/s40520-024-02700-2)
Supplement: Supplementary file 1 — Supplementary file1 (DOCX 201 KB) [file 40520_2024_2700_MOESM1_ESM.docx]

## **Appendices**

**Annex A.** Searches

| Database | Search Phrase |
| --- | --- |
| PubMed | (((("Executive Function" OR "Cognitive Flexibility" OR Cognitive) AND (Multidomain OR Multi-domain OR Multicomponent OR "Dual task")) AND (Sarcopenia OR Strength* OR "Resistance training" OR Exercise)) NOT (hospital*[Title/Abstract])) NOT (supplement*[Title/Abstract]). |
| Scopus | ( TITLE-ABS-KEY ( "Executive Function"  OR  "Cognitive Flexibility"  OR  cognitive )  AND  TITLE-ABS-KEY ( multidomain  OR  multi-domain  OR  multicomponent  OR  "Dual task" )  AND  TITLE-ABS-KEY ( sarcopenia  OR  strength*  OR  "Resistance training" )  AND NOT  TITLE-ABS-KEY ( hospital*  OR  supplement* )  AND  TITLE ( randomised  OR  randomized  OR  rct ) )  AND  PUBYEAR  >  2011  AND  Limit to article. |
| Cochrane Central | "Executive Function" OR "Cognitive Flexibility" OR Cognitive in Title Abstract Keyword AND Multidomain OR Multi-domain OR Multicomponent OR "Dual task" in Title Abstract Keyword AND Sarcopenia OR Strength* OR "Resistance training" in Title Abstract Keyword AND aged OR aging or *lder in Title Abstract Keyword NOT hospital* OR "care homes" OR "nursing homes" in Title Abstract Keyword - with Publication Year from 2012 to present, with Cochrane Library publication date from Jan 2012 to present, in Trials (Word variations have been searched). |
| Science Direct | ("Executive Function") AND (Multidomain OR "Dual task" OR multicomponent) AND (Strength OR "Resistance training") AND (Older OR Aging) NOT (supplementation OR Children). |
| PEDro | Simple Search: “Cognitive flexibility” AND Exercise. |
| Nature | "Cognitive flexibility" AND (Sarcopenia OR Strength) AND (Older OR Aging). |
| Web of Science | "Executive Function" OR "Cognitive Flexibility" OR Cognitive (Abstract) AND Multidomain OR Multi-domain OR Multicomponent OR "Dual task" (All Fields) AND Sarcopenia OR Strength* OR "Resistance training" OR Exercise (Abstract) NOT hospital* (Abstract) NOT supplement* (Abstract) AND elder OR aging OR older (Abstract) NOT nutrition* OR supplement* (Abstract) and Article (Document Types). |
| EBSCO | "AB ( "Executive Function" OR "Cognitive Flexibility" OR Cognitive ) AND AB ( Multidomain OR Multi-domain OR Multicomponent OR "Dual task" ) AND AB ( Sarcopenia OR Strength* OR "Resistance training" OR Exercise ) NOT TX ( Hospital OR supplement* ) Full Text; Publication Year: 20120101- AND apply to Equivalent Materials”. |

| **Annex B - Table 1.** Study characteristics – Systematic Review | | | | | | | | |  |
| --- | --- | --- | --- | --- | --- | --- | --- | --- | --- |
| **Author** | **Country** | **Experimental Group.** | | **Control Group** | | | **Age (Mean ± SD)** | |  |
| (Adcock et al., 2020) * | Switzerland | n=15  10 female (66.7%) | | | n=16  6 female (37.5%) | | | EG. 77.0±6.4 // CG. 70.9±5.0 | |
| (Bae et al., 2019) | Japan | n= 41  18 female (43.9%) | | | n= 42  22 female (52.4%) | | | EG. 75.5±6.0 // CG. 76.4±5.1 | |
| (Callisaya et al., 2021) * | Australia | n = 44  27 female (61.4%) | | | n = 49  27 female (55.1%) | | | EG. 72.9±7.2 // CG 72.8±6.9 | |
| (Coelho-Júnior et al., 2021) | Brazil | n = 22  LSRT = EG (11)  9 female (81.8%)  HSRT = EG2 (11)  11 female (100%) | | | n = 10  10 female (100%) | | | EG 65±3.5 // EG265±2.8 // CG 65±3.2 | |
| (Oliveira Silva et al., 2019) * | Brazil | n = 7  6 female (85.7%) | | | n = 12  5 female (41.7 %) | | | EG. 71.8±5.7 // CG. 78.2±5.3 | |
| (Hiyamizu et al., 2012) * | Japan | n = 17  10 female (60%) | | | n = 19  16 female (84%) | | | EG. 72.9±5.1 // CG. 71.2±4.4 | |
| (Hong et al., 2018) | South Korea | n = 12  10 female (83.3%) | | | n = 13  6 female (46.2%) | | | EG. 76±5.2 // CG. 73.2±5.3 | |
| (Huang et al., 2021) | Japan | AT = EG. (92); 49 female (47.1%)  RT = EG2 (95); 49 female (48%)  AT+RT = EG3 (98);43 female (41.3%) | | | | n = 105  54 female (51.4%) | | EG. 72.3±4.6 // EG2. 72.3±4.8 // EG3. 72.6±4.5 // CG. 72.1±4.6 | |
| (Iuliano et al., 2015) | Italy | AT = EG. (20)  12 female (60%)  RT = EG2. (20)  11 female (55%) | | | n = 20  12 female (60%) | | | EG. 68.4±6.4 // EG2. 65.8±6.32 // CG. 68.44±6.32 | |
| (Kang et al., 2014) | South Korea | n = 14  14 female (100%) | n = 7  7 female (100%) | | | | | EG. 75±3.5 // CG 78±2.8 | |
| (Kim et al., 2021) * | South Korea | n = 10  8 female (80%) | n = 10  9 female (90%) | | | | | EG. 68.3±6.3 // CG. 64.7±6.8 | |
| (Liang et al., 2021) | Taiwan | n = 30  12 female (60%) | n = 57  39 female (68.4%) | | | | | EG. 70.3±4.6 // CG. 70.5±4.4 | |
| (Nishiguchi et al., 2015) * | Japan | n = 24  11 female (45%) | n = 24  11 female (45.8%) | | | | | EG. 73±4.8 // CG 73.5±5.6 | |
| (Park et al., 2019) * | South Korea | n = 23  15 female (65.2%) | n = 22  15 female (68.2%) | | | | | EG. 70.55±6.4 // CG. 72.7±5.3 | |
| (Rosado et al., 2021) * | Portugal | n = 32  29 female (90.6%) | n = 19  13 female (68.4%) | | | | | EG. 74.6±5.5 // CG. 76.8±6.45.8 | |
| (Wang et al., 2018) | Taiwan | n = 16  4 female (25%) | n = 11  3 female (27.3%) | | | | | EG. 71±5.5 // CG. 70.3±4.7 | |
| (Wollesen et al., 2017) | Austria | n = 23  15 female (65.2%) | n = 26  19 female (73.1%) | | | | | EG. 71.7±4.9 // CG. 73.7±5 | |
| LSRT = Low Speed Resistance Training; HSRT = High Sped Resistance Training; EG = Experimental Group; AT = Aerobic Training; RT = Resistance Training. *Studies included in the Meta-Analysis. | | | | | | | | | |

|  | | | | | |
| --- | --- | --- | --- | --- | --- |
| **Annex C - Table 2.** Study Intervention – Systematic Review | | | | | |
| **Study** | **Intervention** | **Training Volume** | **Dose/Intensity** | **Additional** | |
| (Adcock et al., 2020) * | 16 weeks - Exergame:  1.- Strength-Tai Chi inspired exercises, 2.- dancing, and 3.- step-based cognitive games | 30–40 min  3 session/week  48 sessions | Written recommendations for Moderate intensity.  Not progressive adaptations, nor monitoring. | Biweekly phone calls to both groups. | |
| (Bae et al., 2019) | 24 weeks - Multicomponent:  1.- Physical, 2.- Cognitive, and 3.- social activities; Individually Tailored. | 90 min  2 session/week  48 sessions | Moderate intensity measured with accelerometer | Exercise of personal choice  Small groups.  2 health education classes of 90 mins. | |
| (Callisaya et al., 2021) * | 24 weeks – Exergame/Multicomponent:  1.- Balance, 2.- Strength and 3.- Cognitive activities. Individually Tailored. | 10-30 min / session  40-120 min / week | N/I intensity  Progressive difficulty | Follow up visits and phone calls | |
| (Coelho-Júnior et al., 2021) | 16 weeks – Resistance:  4 resistance exercises | 4-8 sets of 4-10 repetitions | Moderate intensity  N/I in how measured | N/I | |
| (Oliveira Silva et al., 2019) * | 12 weeks - Multicomponent:  1.- Balance, 2.- aerobic, 3.-strength and 4.- stretching activities. | 60 min  2 session/week  24 sessions | Aerobic: 70%VO_2max_ / 80% HR_max_  Strength: 3 sets (8–12rep and 60” rest) | Clinical follow up | |
| (Hiyamizu et al., 2012) * | 12 weeks – Multicomponent:  1.- Strength, 2.- Balance, 3.- Flexibility and 4.- Dual task motor-cognitive. | 60 min  2 session/week  24 sessions | Low-intensity strength  N/I in how measured | N/I | |
| (Hong et al., 2018) | 12 weeks – Resistance: elastic band routine | 60 min  2 sessions/week  24 sessions | 15 RM  Progressive overload | NI | |
| (Huang et al., 2021) | 26 weeks – Aerobic or Strength or Combined. | 60 min  2 session/week  52 sessions | RT: progressive from Easy to Hard in RPE – 2 sets 10 rep.  AT: Heart Rate Monitor 40-70% of HRR | | Home-based self-training, booklet with safety tips &  26 weeks post follow up |
| (Iuliano et al., 2015) | 12 weeks - Aerobic or Strength training | 60 min | 50-80% HRR  60-85% of 1RM / 3 series of 6-12 reps for 6 exercises | | NI |
| (Kang et al., 2014) | 12 weeks – High speed resistance band training | 60 min  1 session/week  12 sessions | 12-13 RPE | | 3/week - Individual work at home |
| (Kim et al., 2021) * | 12 weeks – Exergame/Multicomponent:  1.- Aerobic, 2.- Dual task motor-cognitive (cognitive, speed reaction and strength) | 30 min  2 session/week  24 sessions | Progressive intensity  N/I in how measured  3.5 to 4 km/h for aerobic | | N/I |
| (Liang et al., 2021) | 52 weeks – Education/Multicomponent: exercise, cognitive training, diet education and individualized integrated care for multimorbidity | 120 min  16 sessions/year  16 sessions | N/I | | Education, follow up every 3 months and independent exercise. |
| (Nishiguchi et al., 2015) * | 12 weeks – Multicomponent:  1.- Flexibility, 2.- Strength and 3.- Dual task aerobic-cognitive. | 90 min  1 session/week  12 sessions | ACSM recommendations  N/I in how measured | | Pedometer autonomous walking program. |
| (Park et al., 2019) * | 24 weeks – Dual task aerobic-cognitive | 90 min  1 session/week  24 sessions | Aerobic: 55-80% HR_max_ | | General health education sessions |
| (Rosado et al., 2021)* | 24 weeks – Multicomponent/ Dual task training | 75 min  3 sessions/week  36 sessions | Intended 13 points on the RPE scale | | Additional group with whole-body vibration |
| (Wang et al., 2018) | 12 weeks – Multicomponent: 1.- Resistance, 2.- Endurance, 3.- Balance | 60 min  3 sessions/week  36 sessions | 10 reps at 75-80% Max. Voluntary contraction  70-75% HRR | | Biweekly education sessions |
| (Wollesen et al., 2017) | 12 weeks – Strength and Resistance Training | 60- 90 min  1 session/week  12 sessions | 10-12 reps  Progressive overload  N/I how measured | | N/I |
| VO_2max_ = Maximal oxygen consumption; HR_max_ = Predicted maximum Heart Rate; N/I = No information; RPE = Rate of Perceived Exertion; HRR = Heart Rate Reserve; ACSM = American College of Sports Medicine; *Studies included in the Meta-Analysis. | | | | | |
|  | | | | | |

| **Annex D - Table 3.** Study Results – Systematic Review | | | |
| --- | --- | --- | --- |
| **Study** | **Outcome Measures** | | **Results** |
| (Adcock et al., 2020) * | Trail Making Test (TMT)  Victoria Stroop Test (VST)  30 seconds sit to stand test (30sts)  10 meters walking tests (10mWT) | Post tests.  TMT-A: EG 38 (33.5; 41) // CG 33 (26.5; 47.75) *p* = 0.904  TMT-B: EG 104 (87; 119.5) // CG 91 (71; 108.25) *p* = 0.219  VST-1: EG 14 (13.5; 16) // CG 13 (11.75; 15.5) *p* = 0.963  VST-2: EG 20 (18; 22) // CG 16 (13.75; 21) *p* = 0.650  VST-3: EG 30 (25.5; 34) // CG 27.5 (22.25; 35.25) *p* = 0.046  30sts: EG 13 (13.0; 15.5) // CG 15.5 (12.75;18.75) *p* = 0.024  10mWT (ST): EG 1.29 (1.17; 1.38) // CG 1.40 (1.31; 1.46) *p* = 0.904  10mWT (DT): EG 1.13 (1.06; 1.27) // CG 1.30 (1.22;1.36) *p* = 0.518 | |
| (Bae et al., 2019) | TMT  Handgrip strength (HGS)  2.4 walking test (2.4 WT) | Mean difference pre-post.  N/R | |
| (Callisaya et al., 2021) * | 6.4 walking test (6.4 WT)  5 sit to stand test (5STS)  TMT  VST | Post tests. *p*  <0.05  6.4 WT: EG 1.30 (CI 95% 0.00, 0.01) // CG. 1.21 (CI 95% 0.04, 0.02)  5STS: EG 11.9 (CI 95% 0.8, 2.2) // CG 9.7 (CI 95% 0.8, 2.2)  TMT-A: EG 31.9 (CI 95% 5.8, 0.2) // CG 33.3 (CI 95% 3.7, 2.7)  TMT-B: EG 76.9 (CI 95% 10.4, 7.7) // CG 92.2 ( CI 95% 8.4, 12.2)  VST-1: EG 13.2 (CI 95% 1.7, 0.1) // CG 14.9 (CI 95% 1.1, 0.7)  VST-2: EG 19.0 (CI 95% 1.2, 0.6) //CG 18.7 (CI 95% 1.6, 0.4)  VST-3: EG 31.2 (CI 95% 4.5, 3.6 ) // CG 35.5 (CI 95% 2.9, 6,1) | |
| (Coelho-Júnior et al., 2021) | VST  Timed Up and Go (TUG)  5STS  HGS  4 m Walking Test (4WT) | Post tests.  VST-1: EG 11.19 ±0.77 // EG2 8.19±1.18 // CG 8.31±1.84 *p* < 0.05  VST-2: EG 10.22±1.08 // EG2 9.02±0.98 // CG 9.12±1.88 *p* < 0.05  VST-3: EG 6±1.44 // EG2 3.44±1.04 // CG 0.74±0.22 *p* < 0.05  TUG: EG 6.1±1 // EG2 7.8±2.1 // CG 6±1.1 *p* < 0.05  5STS: EG 6.6±0.8 // EG2 7.3±2 // CG 9±1.1 *p* < 0.05  HGS-R: EG 27.4±5.3 // EG2 20.7±4 // CG 25.7±2.9 *p* < 0.05  HGS-L: EG 27.3±6.3 // EG2 20±4.9 // CG 25.6±3 *p* < 0.05  4WT: EG 1.8±0.3 // EG2 1.4±0.2 // CG 2.21±0.4 *p* < 0.05 | |
| (Oliveira Silva et al., 2019) * | VST  8-foot Time Up and Go (8TUG) | Post tests.  VST-3: EG. 32.83 ± 8.23 // CG. 38.06 ± 11.99 *p* = 0.11  8TUG: EG. 5.6 ± 1.2 // CG. 6.5 ± 1.1 *p* = 0.03 | |
| (Hiyamizu et al., 2012) * | 30 seconds Sit to Stand (30STS)  TUG  TMT  VST | Post tests.  30STS: EG. 21.47 ± 5.93 // CG. 22 ± 5.77 *p* = 0.78  TUG: EG. 6.82 ± 1.78 // 6.91 ± 1.06 *p* = 0.86  TMT-A: EG. 48.63 ± 19.22 // 60.15 ± 39.33 *p* = 0.28  TMT-B: EG 115.89 ± 45.36 // 136.45 ± 78.73 *p* = 0.35  VST-3: EG. 26.23 ± 8.07 // CG. 24.57 ± 11.35 *p* = 0.17 | |
| (Hong et al., 2018) | 8TUG  VST | 8TUG: EG. 5.4±0.47 // CG. 5.71±1.37 *p* < 0.01  VST-2: EG. 23±6.38 // CG. 21.85±5.22 *p* = 0.37  VST-3: EG. 19.42±6.64 // CG. 19.15±4.07 *p* = 0.343 | |
| (Huang et al., 2021)* | HGS  Walking test  TMT | Mean difference pre-post.  Estimated effect per domains, not outcome measures.  N/R | |
| (Iuliano et al., 2015) | VST  TMT  1RM muscle groups | *p* < 0.05  VST-3: EG1. 22.7± 7.3 // EG2. 23.1±9.7 // CG. 24.6±11.8  TMT-A: EG1. 40±13.2 // EG2. 35.1±8.1 // CG. 39.2±14.3  TMT-B: EG1. 66.7±22.3 // EG2. 69.9±21.9 // CG. 79.6±35.2 | |
| (Jeong et al., 2021) | Lean body mass (LBM)  Muscle mass (MM)  TMT | Post tests.  LBM: EG 40.41± 8.62 // 40.68 ± 6.17 *p* = 0.88  MM: EG 37.66 ± 8.05 // 37.92 ± 5.72 *p* = 0.87  Measured by second per letter.  TMT-A: EG. 2.01 ± 0.95 // CG 2.42 ± 1.41 *p* < 0.05  TMTB: EG. 2.40 ± 1.18 // CG 3.17 ±1.25 *p* = 0.01 | |
| (Kang et al., 2014) | MM  % Fat  VST  SPPB  TUG | MM: EG. 18.8±2.8 // CG. 17.1±1.15 *p* = 0.25  %Fat: EG. 38.8±5.4 // CG. 33.4±8.3 *p* = 0.24  VST-2: EG. 13.57±6.17 // CG. 10.14±3.76 *p* > 0.05  VST-3: EG 10.71±3.22 // CG. 6.86±2.19 *p* = 0.03  SPPB: EG. 10.79±1.59 // CG. 7.57±0.98 *p* = 0.01  TUG: EG. 9.14±1.42 // CG. 10.59±1.03 *p* > 0.05  HGS: EG. 24.01±4.14 // CG. 18.99±1.81  *p* < 0.05 | |
| (Kim et al., 2021) * | MM (kg)  % Fat  SPPB  TUG  HGS  Korean Stroop Test (KST) | Post tests.  MM: EG. 21.35 ± 2.33 // CG. 10.44 ± 2.53 *p* < 0.05  %Fat: EG. 36.04 ± 6.71 // CG. 30.34 ± 6.65 p = 0.09  SPPB: EG. 11.8 ± 0.63 // CG. 11.8 ± 0.63 *p* = 0.11  TUG: EG. 5.79 ± 0.94 // CG. 5.8 ± 0.91 *p* = 0.54  HGS-R: EG. 24.2 ± 5.86 // CG. 23.1 ± 3.87 *p* = 0.93  HGS-L: EG. 21.62 ± 7.63 // CG. 20.38 ± 3.51 *p* = 0.89  KST: EG. 93.70 ± 22.35 // 96.8 ± 22.63 *p* = 0.70 | |
| (Nishiguchi et al., 2015) * | TMT  TUG  WT (m/s)  5STS | Post tests.  TUG: EG. 6.54 ± 1.02 // CG. 6.32 ± 1.17 *p* = 0.70  5STS: EG. 6.88 ± 1.26 // CG. 7.85 ± 2.14 *p* = 0.002  TMT-B: EG. 30.4 ± 16.1 // CG 41.5 ± 30.7 *p* = 0.02  WT: EG. 1.40 ± 0.19 // CG. 1.27 ± 0.13 *p* = 0.004 | |
| (Park et al., 2019) * | WT(m/s)  TMT  HGS  TUG  5STS | Post tests.  WT: EG. 1.11 ± 0.4 // CG 1.05 ± 0.3 *p* = 0.02  TMT-A: EG. 23.1 ± 6.3 // CG. 24.1 ± 6.7 *p* = 0.1  HGS-R: EG. 28.6 ± 5.4 // CG. 29.0 ± 6.8 *p* = 0.132  TUG: EG. 8.9 ± 3.4 // CG. 9.5 ± 3.9 *p* < 0.01  5STS: EG. 16.9 ± 4.5 // CG. 17.7 ± 4.8  *p* < 0.01 | |
| (Rosado et al., 2021) * | TUG | Post tests.  TUG: EG. 390.9 ± 77.3  *p* = 0.444 // CG. 460.5 ± *p* = 0.104 | |
| (Wang et al., 2018) | C-EXIT 2  Gait-cognitive | Outcome measures not used in other studies.  N/R | |
| (Wollesen et al., 2017) | VST  Gait - Step length | VST-3: EG 11.7 (CI 95% 13.3, 9) // CG. 11.7 (CI 95% 13.5, 9.1)  *p* > 0.05 | |

Trail Making Test = TMT; Victoria Stroop Test = VST; 30 seconds sit to stand test = 30sts; 10 meters walking tests = 10mWT (expressed in m/s); EG = Exercise Group; CG = Control Group; 2.4 WT = 2.4 walking test; WT = walking test ; N/R = not relevant; 5STS = 5 sit to stand test; 8TUG: 8 foot Time Up and Go; TUG: Timed Up and Go; 4WT = 4m Walking Test; 30STS = 30 seconds Sit to Stand Test; KST: Korean Stroop Test; MM= Muscle Mass; SPPB= Short Physical Performance Battery; HGS= Handgrip Strength. *Studies included in the Meta-analysis.

**Annex E.** TESTEX methodological risk


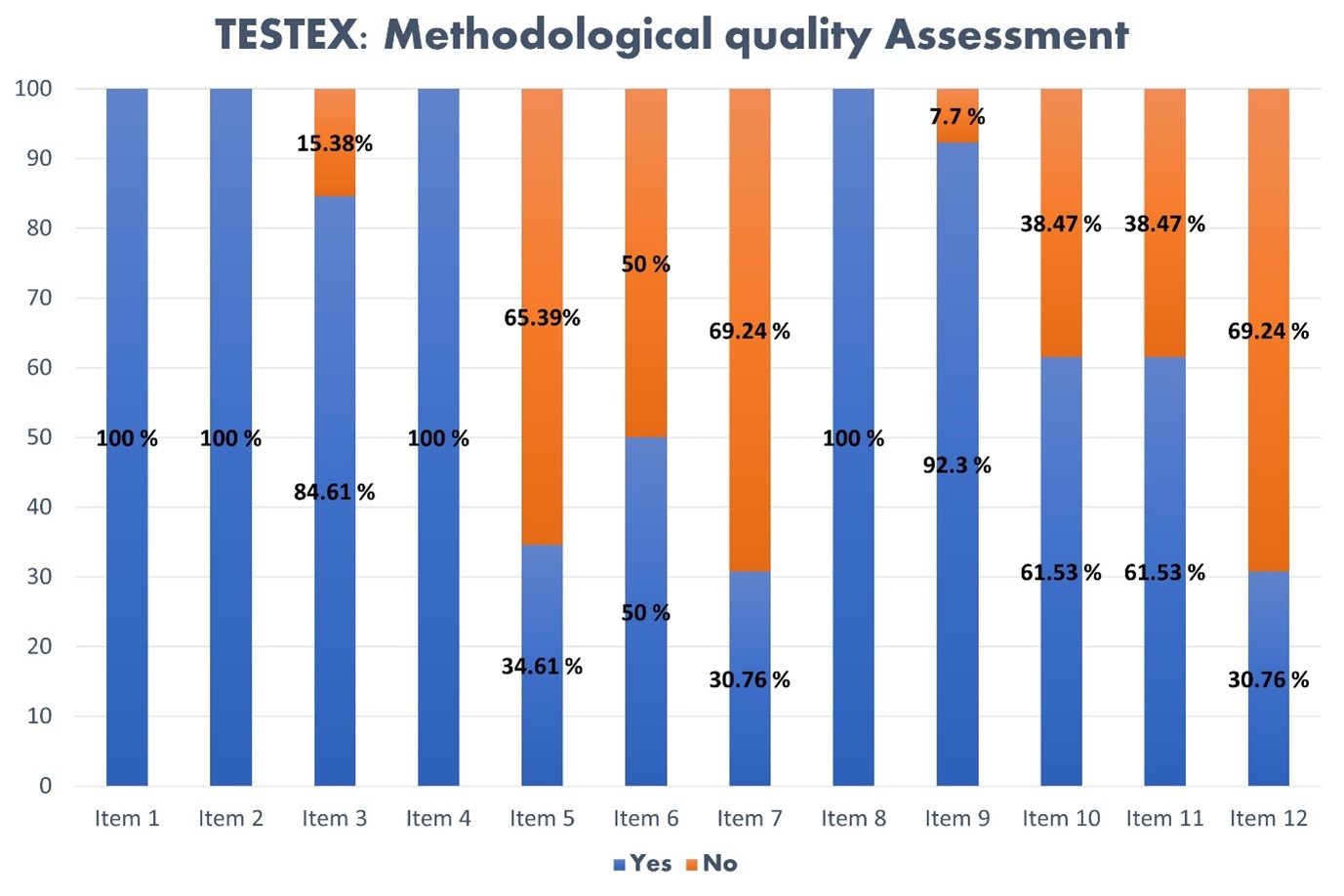


**Annex F.** Level of evidence (GRADE)

| **Certainty assessment** | | | | | | | **№ of patients** | | **Effect** | | **Certainty** | **Importance** |
| --- | --- | --- | --- | --- | --- | --- | --- | --- | --- | --- | --- | --- |
| **№ of studies** | **Study design** | **Risk of bias** | **Inconsistency** | **Indirectness** | **Imprecision** | **Other considerations** | **Exercise** | **No exercise** | **Relative (95% CI)** | **Absolute (95% CI)** |  |  |
| **TUG** | | | | | | | | | | | | |
| 6 | randomised trials | not serious | not serious | not serious | not serious | none | 115 | 118 | - | SMD **0.25 SD lower** (0.51 lower to 0.01 higher) | ⨁⨁⨁⨁ High | IMPORTANT |
| **TMT** | | | | | | | | | | | | |
| 3 | randomised trials | not serious | not serious | not serious | not serious | none | 58 | 57 | - | SMD **0.38 SD lower** (0.75 lower to 0) | ⨁⨁⨁⨁ High | IMPORTANT |
| **STS** | | | | | | | | | | | | |
| 3 | randomised trials | not serious | not serious | not serious | not serious | none | 91 | 95 | - | SMD **0.34 SD lower** (0.63 lower to 0.05 lower) | ⨁⨁⨁⨁ High | IMPORTANT |
| **VST** | | | | | | | | | | | | |
| 4 | randomised trials | not serious | not serious | not serious | not serious | none | 98 | 92 | - | SMD **0.02 SD higher** (0.27 lower to 0.3 higher) | ⨁⨁⨁⨁ High | IMPORTANT |

Confidence interval = CI; Standardised mean difference = SMD.
